# A mixed-method analysis to identify the current focus, trends, and gaps in health science research in Saudi Arabia

**DOI:** 10.3389/fpubh.2022.1028361

**Published:** 2023-01-13

**Authors:** Asma Ayyed AL-Shammary, Sehar un-Nisa Hassan, Fahad Saud Alshammari, Modi Rataan Rifai Alshammari

**Affiliations:** ^1^Department of Public Health, College of Public Health and Health Informatics, University of Ha'il, Ha'il, Saudi Arabia; ^2^Department of Health Management, College of Public Health and Health Informatics, University of Ha'il, Ha'il, Saudi Arabia; ^3^Department of Health Informatics, College of Public Health and Health Informatics, University of Ha'il, Ha'il, Saudi Arabia

**Keywords:** medical research, gaps, trends, health research, Saudi Arabia, mixed-method analysis

## Abstract

**Background:**

The identification of current gaps in high-impact medical research in Saudi Arabia has international significance due to the trend of collaborative research in the field of health and medicine and the focus on knowledge-sharing. The purpose of this study is to assess the current focus, gaps, and priorities in health research in Saudi Arabia.

**Methods:**

We employed a mixed-method research approach to achieve research objectives. (1) a systematic review of scientific research studies that are published between January 2020 to January 2022 in the top fifty Q1 medical science journals (2) a cross-sectional survey collected data from professionals employed in various organizations including the Ministry of Health (MoH), Ministry of Education (MoE), health organizations and universities, and the health industry. The close-ended survey questions inquired about the broad and specific areas of ongoing health research projects by these researchers and organizations in Saudi Arabia.

**Results:**

The literature search on databases identified Science Direct (*n* = 741), Pub Med (*n* = 244) and Google Scholar (*n* = 15,600). After screening, (*n* = 26) original studies were selected for detailed evaluation and synthesis. Among these (*n* = 7) studied infectious diseases, (*n* = 7) cancer, and cardiac disease (*n* = 5). These studies focused on the etiology, treatment management and therapy outcomes of these health conditions. The survey was completed by (*n* = 384) respondents from these organizations. Most of the ongoing research projects focus on clinical sciences (27%) followed by basic sciences (24%) and public health research (24%) and a limited number of researchers were involved in healthcare management (2%) and informatics (2%). Most research focused on kidney and liver disorders (80%), obesity (74%), diabetes (74%), hormonal diseases (64%), and infectious disease (66%); it is equally important to design and fund research in some of the neglected areas including reproductive health (3%), physical and mental disabilities (1%).

**Conclusion:**

Findings suggest that current gaps in original research from Saudi Arabia are in healthcare service quality, reproductive health, physical and mental disabilities and health informatics. Researchers and funding agencies and international collaborative projects should prioritize these areas.

## 1. Introduction

The World Health Organization (WHO) urges global, regional, and local research to improve access and delivery of healthcare services to all sections of the population in accordance with their regional and national circumstances (1). The WHO's strategy for research on health is based on the claim that good quality scientific knowledge should be used to formulate policies and practices to promote global health. Health organizations and academic institutions in both developed and developing countries have focused on developing more human resources through research training. In recent times, local and international funding organizations have invested more funds in health research.

Research studies in the field of health sciences assess health problems, understand their causes, utilize the research findings to formulate policies, practices, and products; and evaluate the effectiveness of health interventions. Saudi 2030 vision also aims to devise efficient healthcare systems by expanding and improving the quality of healthcare services as well as financing health services to achieve universal health coverage. The Saudi government allocated both financial and human resources in scientific research to strengthen the procedures for data sharing and access to updated information and supported institutions and health organizations to conduct research, which is helpful in assessing their progress toward the Saudi 2030 vision. In 2015, more than 3.2 billion Saudi Riyal were invested in science and technology projects, out of which ~23% of the total funding went to medical and health research projects. In addition, the Ministry of Education (MoE) has taken nearly 24 initiatives to support research and development activities including research capital funding, university-industry partnership programs, international collaborations, research training, and knowledge-sharing platforms (2). Researcher organizations and researchers working on international collaborative projects need valid information to understand the existing gaps in health research to set the direction for future research projects and enhance the research output. It is, therefore, pertinent to assess the research outcomes, current focus, and future trends in health research in Saudi Arabia to monitor progress toward the research and development milestones of the Saudi 2030 vision.

Health research includes both healthcare and health services research, which relates to various fields, including life sciences, medical, psychological, social sciences, health informatics, and management sciences (3). The term “health research” was taken to cover both primary and secondary research, including clinical trials, field studies, cross-sectional surveys, community-based intervention studies, analyses of existing demographic and health data to develop health policies, and analytical research including systemic reviews and meta-analyses (4). According to the WHO (5) the guiding principle for successful health research includes the features of quality, impact, and inclusiveness. Good quality health research is concise in methodology, unbiased, ethical, carefully monitored and evaluated for adherence to procedures, and provides an accurate interpretation of the findings. The impact of health research is determined by its capacity to improve local and global health outcomes, address health inequities, and foster health-related development. Health research focuses on inclusiveness through collaboration between academia and industry and mainstream research by engaging the community in the research process. All these features make it possible to achieve the desired outcomes of health research.

Research productivity in the field of health sciences in the Saudi Arabia has been estimated by only a few previous studies. A bibliometric analysis of scientific research studies published between period of 2008–2017 were retrieved from the Scopus Database (6). A total of 35,291 articles were retrieved, with the highest number of publications during 2017, demonstrating rising trends in research output between 2008 and 2017 and a promising growth rate of 59% between 2014 and 2017. This review identified gaps in research in the fields of nursing and neuroscience. The major contributor among academic institutions for health research was King Saud University Saudi Arabia as demonstrated in previous research (7). The outcomes of initiatives taken by other institutions to improve research productivity in the past few years are not known.

A bibliometric analysis of research papers that were published between 1980 and 2014, demonstrated that (33%) of research articles published during this period were focused in area of clinical and health research (8). A review of health research published between 1996 and 2012 found 27,246 papers of which only 151 belonged to high-impact factor journals (9). A previous study from Saudi Arabia assessed biomedical research productivity between 1982 and 2000 by conducting another bibliometric analysis (10). A total of (*n* = 5,962) research articles were published in PubMed indexed journals from Saudi Arabia during this period. Out of total research that was contributed by countries from Arab sphere during this period, around 27% was by Saudi Arabia.

The current reliable estimates of high-impact research productivity in Saudi Arabia are limited to scholarly research published until 2017 (6, 11). There is one recent study which examined trend of international collaborations for research in Saudi Arabia between 2001 to 2022 and found that due to collaborative projects the average annual growth rate of scientific publications in Saudi Arabia was raised to 17% (12). Both these studies are based on bibliometric analysis and lacking the input/opinion of all stakeholders.

The current study adopts a mixed-method approach to gain deeper insight that will be useful for setting appropriate courses of action to achieve the research and development goals. The identification of current gaps in high-impact medical research in Saudi Arabia has international significance due to the trend of collaborative research in the field of health and medicine and focus on knowledge-sharing. According to recent estimates, 65% of Saudi Arabia's scientific output between in the past two decades involved international collaborations (12). International research organizations and researchers located in various countries could use the insight from this research to design appropriate proposals for international collaborative research projects.

The specific objectives of this study are as follows:

- To assess the current trends and existing gap in health science research in Saudi Arabia.
- To determine future priorities for scientific research in health science.
- To make suggestions for the re-formulation of health research strategies and international collaboration in specific area to enhance high-impact research productivity in Saudi Arabia.

## 2. Methods

### 2.1. Study design

The study employed a mixed-method research approach that included (1) a systematic review of high-impact scientific research studies published between January 2020 and January 2022 in the top fifty Q1 journals and (2) a cross-sectional survey of key stakeholders comprising researchers, administrators and key informants in organizations that fund clinical and public health research in Saudi Arabia conducted in March and April 2022.

### 2.2. Systematic review of high-impact original research in medicine from Saudi Arabia

The systematic review was completed using the PSALSAR framework which has six steps. The details of each step followed to complete this systematic review are presented in Table 1.

**Table 1:** PSALSAR framework utilized to conduct a systematic review of health research studies in Saudi Arabia.

**Steps**	**Strategy and expected outcome**	**Methods**
Protocol search	*Study scope*: Review the high-impact health research produced from Saudi Arabia and original research studies published in the top fifty Q1 journals in the field of medicine in the past 2 years	Evaluating the type of article, the topic of study, the target population, the study outcome and the funding source
	*Search strategy*: Searching selected databases covering health science research	Databases used in the search included Science Direct, Pub Med and Google Scholar The search string was completed by using the title, abstract, and keywords The keywords used were “Saudi Arabia” AND “Health” AND “Disease”
Appraisal	*Evaluation of studies:* Selecting studies and evaluation of studies against the indicators developed according to research questions The published evaluated to dig out information about *a. Topic of study*: Original studies that focus on any health issues or healthcare in Saudi Arabia *b. Study population:* The study should be conducted in the Saudi population *c. Nature of comparison:* Study that compares or adaptation of treatments or health programs d. *Type of health or intervention outcome*: How the gaps in knowledge and research reported in these publications e. *Context of study:* The study settings	*The inclusion criterion:* Published research in the past 2 years between January 2020 and Apr 2022 in the top 50 Q1 journals in the area of Medicine [SCIMAGO (13)]. Quantitative research, randomized and non-randomized clinical trials, descriptive and cross-sectional studies, clinical population, community population, communicable and non-communicable diseases, disability, public health, pilot projects for intervention studies *The exclusion criterion:* Systematic review, review articles, studies that do address science aspects but are not focused on health or medical sciences, studies on animals or plants, articles published before January 2020
Synthesis	Data extraction Categorization of data	Data extraction template were used and data was extracted independently by researchers Excel file templates were used to categorize the data
Analysis	Data analysis Results and discussion Conclusion	The analysis is completed to show the focus and gaps in high impact research from Saudi Arabia in field of medicine Making suggestion based upon key insights to address gaps and priorities for future research
Write up of manuscript	Methodology writing	^*^PRISMA methodology
Publishing	Journal Article format	Preparing in accordance with journal systematic review paper requirements

* PRISMA, preferred reporting items for systematic reviews and meta-analyses.

### 2.3. Online survey method

#### 2.3.1. Survey respondents

Data on the online survey form were collected from key informants and researchers working in various organizations in Saudi Arabia during the study period. These included administrators from the Ministry of Health (MoH) and the Ministry of Education (MoE), researchers and faculty from higher educational institutions/universities, governmental and non-governmental healthcare organizations, research funders, independent researchers in health organizations and universities, research consultants, staff in health industry involved in research and development projects. A total of (*n* = 384) professionals who were in any of the three given roles, namely a researcher, an administrator, or a representative of the funding organization/s, completed the survey forms.

#### 2.3.2. Survey questionnaire

The survey questionnaire comprised three parts. The first part provided information about the study, assessed the prospective participant on the study inclusion/exclusion criteria, and obtained informed consent. Those who met the inclusion/exclusion criteria and consented to participate proceeded to the next screening of the survey form. In the second part of the survey, respondents provided information about their affiliation with the target organization from the given list of organizations and the current work role that relates with scientific research. The third part of the survey obtained information about the broad areas and specific types of health problems being studied in the respondents' ongoing research projects. The respondents were required to provide a yes or no response to all the areas and topics of health research presented in the survey form. A pilot survey was completed for the pre-testing of survey items to ensure their appropriateness. Both the English and Arabic versions of the survey form were presented, and respondents could choose their preferred language to complete the survey.

### 2.4. Study procedures

For a systematic review, the search was completed by two researchers, and data extraction was completed by two researchers independently using data extraction forms. The information was categorized into MS Excel sheets for synthesis and analysis. Data collection for the cross-sectional online survey was completed in March 2022 and April 2022. The survey was distributed using a Google Forms link, which was shared with the target research community through researchers and health professionals' online communities.

### 2.5. Data analysis

The synthesis and analysis of data from the systematic review were completed by following the PRISMA methodology, and the findings are presented accordingly using percentage values that match the descriptive nature of the study. The data were entered and analyzed with IBMSPSS v.25 and graphs were made using MS Excel.

### 2.6. Ethical considerations

The authors of this study adhered to the research ethics guidelines of the Heliniski Code of Conduct. Informed consent was obtained from the research participants, and the anonymity of the data was maintained at all stages of research in accordance with the ethical principles.

## 3. Results

### 3.1. Findings from systematic review of published research

**Figure 1:**
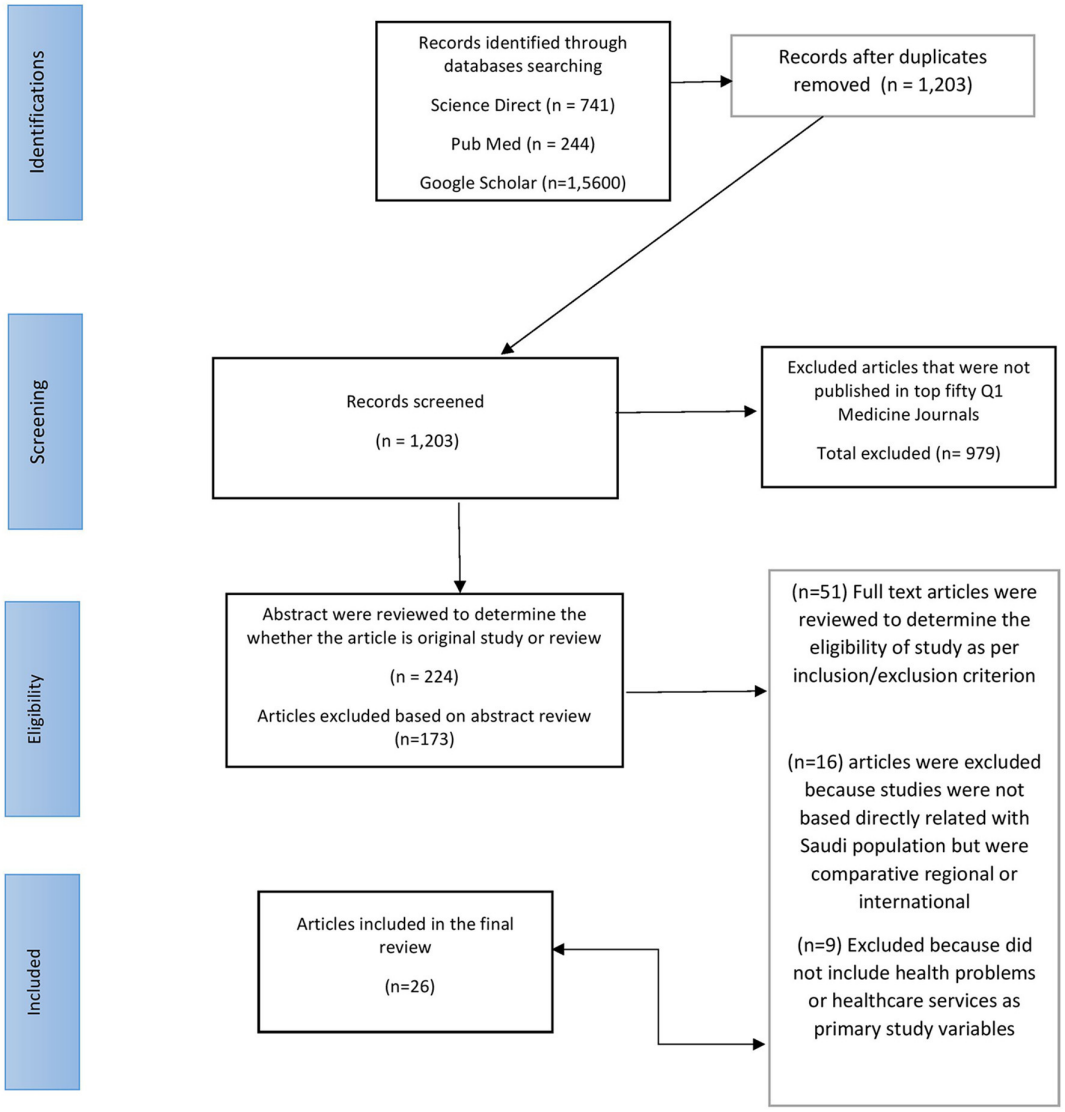
Preferred reporting items for systematic reviews and meta-analyses (PRISMA) flow chart.

A total of 16,585 articles were retrieved from two rounds of literature search that were completed in January 2022. The flowsheet diagram shows the process of article search and evaluation at different levels, including those published in target journals, topics, abstracts, methodologies, and populations (Figure 1).

The information extracted from the articles published in the top 50 Q1 medicine journals between January 2020 and January 2022 is shown in Table 2. It was observed that most of the studies published by research teams from this country were systematic review, metanalysis or not focused on human health conditions. Overall, a low number of original (*n* = 26) studies from Saudi Arabia were published in these journals during this period. Out of (*n* = 26) original studies, (*n* = 7) focused on infectious diseases, cancer (*n* = 7) cardiac disease (*n* = 5) particularly the etiology, treatment management, and therapy outcomes of these diseases. Other published studies focused on diabetes (*n* = 1), dental problems (*n* = 1), maternal health (*n* = 1), child obesity (*n* = 1), inherited disease condition (*n* = 1), emergency healthcare services (*n* = 1), and treatment protocol for amiodarone therapy (*n* = 1). Most of the published studies were funded by key organizations supporting scientific research in Saudi Arabia including the King Abdullah International Medical Research Center, King Abdullah bin Abdul-Aziz University, and some in collaboration with international funding agencies. Pharmaceutical research was funded by pharmaceutical companies such as Pfizer, AstraZeneca and, Genomic Health.

**Table 2:** Original research studies included in review published in top 50 Q1 journals between January 2020 to January 2022 (*n* = 26).

	**References**	**Year**	**Area of study**	**Aim of study**	**Study design and data collection**	**Study sample and size**	**Key insights**
1	Ebrahim et al. (14)	2021	Infectious diseases (MERS-COV)	To determine the epidemiology, laboratory and clinical characteristic, and survival patterns of MERS-CoV	Health records of patients admitted to one of the four MERSCoV referral hospitals in Saudi Arabia, between 2014 and 2019	Suspected (6,873) or confirmed with MERS-CoV(501)	MERSCoV remains the coronavirus with the highest severity (29%) and case fatality rate (21%) among the three lethal coronaviruses
2	Tyrovolas et al. (15)	2020	All diagnosable disease conditions and injury	To describe the levels and temporal patterns in fatalities, ill health, risk factors, healthcare access after healthcare reforms in Saudi Arabia	Descriptive research	Dataset of GDB study conducted in 2017	DALYs due to cardiac diseases (ranked first) and musculoskeletal disorders (ranked second) and transport injuries (ranked third). YLDs due to mental disorders, neurological disorders and substance use disorders steadily increased
3	Alsohime et al. (16)	2020	Inherited disease	Treatment effectiveness	Case study of child with inherited USP18 deficiency	Single case of neonate	Treatment with ruxolitinib has positive treatment outcomes
4	Arabi et al. (17)	2020	Infectious diseases (MERS-CoV)	Treatment outcome for MERS-CoV	Experimental study	In-patients with MERS-CoV	Recombinant interferon beta-1b and lopinavir–ritonavir led to lower mortality than placebo among patients
5	Jenkins et al. (18)	2021	Cardiac diseases	To determine the relationship between the glycemic index and heart disease	Comparative study with longitudinal research design	(*N* = 137,851) participants between the ages of 35 and 70 years living on five continents	A diet with a high glycemic index is related with an increased threat of cardiovascular disease and mortality
6	Alshukairi et al. (19)	2021	Infectious diseases (MERS-CoV)	Studied the antibody responses in MERS-CoV infection survivors	Experimental longitudinal study	48 MERS-CoV survivors from five hospitals	MERS-CoV–specific neutralizing antibodies were detected for 6 years post infection.
7	Alanazi et al. (20)	2020	Infectious diseases Comorbid disease with MERS-CoV	To determine risk of mortality in infected patients with MERS-CoV	Descriptive study	32 virus-infected patients	Disease severity and mortality risk associated with multiple and moresevere underlying conditions
9	Alhazzani et al. (21)	2022	Infectious diseases (COVID-19)	Studies the impact of prone positioning in patients with COVID-19	Randomized clinical trial	400 adults with acute hypoxemic respiratory failure from COVID-19	No difference was found regarding impact of prone position on endotracheal intubation in adults who were awake and not intubated and who had hypoxemic respiratory failure
10	Iskedjian et al. (22)	2020	Cancer	To generate health utilities estimates for health states associated with cancer	Comparative research	Equal number of patients with and without cancer (*n* = 398)	This study provided useful insights about health utility measures for various types and stages of cancer in Saudi Arabia
11	AlAzmi et al. (23)	2020	Healthcare services for cancer patients	To investigate the challenges faced by Saudi Arabia in cancer therapies	Qualitative research	(*n* = 26 pharmacists and *n* = 29 physicians)	Cancer drug shortage is a significant problem in all health centers in Saudi Arabia
12	Jazieh et al. (24)	2020	Healthcare services to handle MERS-COV	Description and impact of the crisis management plan to manage patients with cancer during a MERS-CoV epidemic in Saudi Arabia	Descriptive	Oncology department of hospital	Plan resulted in the prevention of any new infection, evidenced by zero cases of in-hospital transmission of MERS-CoV infection among oncology patients, during and after crisis period
13	Jazieh et al. (25)	2020	Lung cancer	To study the patterns of PD-L1 expression and cluster of differentiation 8 (CD8) immunotoxins in patients with NSCLC	Descriptive cross-sectional study	(*n* = 200) patients with NSCLC were included in the study from six centers in Saudi Arabia and Algeria	Increase the awareness of oncologists in the region about the use of bio-markers and, most importantly, use of checkpoint inhibitors in lung cancer patients
14	Badran et al. (26)	2020	Treatment efficacy of cancer	Efficacy of first-line therapy with sunitinib in patients with mRCC	Medical Records used for retrospective analysis	Fifty-five patients who received sunitinib were identified	Sunitinib results in lower progression-free survival in the studied group compared to published western data
15	Jazieh et al. (27)	2020	Infectious diseases (MERS-COV)	Features of oncology patients with confirmed MERS-CoV	Cases were followed up Descriptive study	19 patients admitted at the Ministry of National Guard Health Affairs-Riyadh	MERS-Covid infection resulted in a high case fatality rate in patients with malignancy
16	Salama et al. (28)	2021	Quality of healthcare services for cancer patients	To assess futile acute care services (ACSs) for patients with cancer	Expert review of medical records of deceased patients	135 patients	Timely documentation of the GOCs for patients with a palliative intent increased significantly from 59% at baseline to 83% in the postintervention phase
17	AlSaleh et al. (29)	2021	Treatment response for cancer disease	Testing the feasibility of 21-gene essay on patients with relative risk	Experimental study	258 patients with relative risk	21-gene assay on biopsies is feasible
18	Siraj et al. (30)	2020	Colorectal cancer	To study mutational mechanism in familial adenomatous polyposis	Descriptive/Lab testing to study mutational mechanism	1,207 CRC patients	A strong founder effect and an unusual mutational mechanism in familial adenomatous polyposis
19	Al-Qahtani et al. (31)	2021	Emergency healthcare	Characteristics of pediatric ER visits, the rate of hospital admissions and its associated predictors	Retrospective, medical record-based study at King Fahd Hospital of the University	Medical records of (*N* = 46,374) out of which (*n* = 1,134) cases admitted	High number of non-urgent ER visits. This further underlines the problems with improper use of ER services
21	Baraka et al. (32)	2021	Pharmacy & maternal health	Safety and use of commonly prescribed antimicrobial drugs during pregnancy	Retrospective study by using medical records	(*n* = 344) pregnant women	High proportion of antimicrobials prescribed during pregnancy that might pose risks to mothers and their fetuses
22	Al-Khalifa et al. (33)	2021	Dental health	compare the ability of the two models to predict dental age in Saudi children	Retrospective medical records	(*n* = 1,146) panoramic radiographs from healthy Saudi children (605 male, 541 female)	Data from the Willems BC population can be used as a reference in the Saudi population
23	Al Slamah et al. (34)	2020	Diabetes	Risk factors Type 2 diabetes self-management to the Saudi context	Secondary data analysis of National Health Survey	(*n* = 808) participants had type 2 diabetes	Being overweight and/or hypertensive are associated with type 2 diabetes in Saudi Arabia
24	Albassam et al. (35)	2020	Treatment protocols	To assess the presentation of the baseline tests before starting amiodarone therapy and the on-going observations	A retrospective descriptive charts review study	143 eligible participants on amiodarone therapy	Need improvement to adapt to local context
25	Alatmi et al. (36)	2020	Cardiac diseases	To describe impact and patterns of possibly harmful medication use in patients with specific cardiac health conditions	A retrospective descriptive charts review study of patients with HFpEF vs. HFrEF patients	200 patients in each group placed after matching according to their age	Validated inapt medication use among patients with HFpEF
26	Al-Hassnan et al. (37)	2020	Child cardiac health and obesity	Genetic cause of childhood-onset cardiomyopathy	Retrospective analysis of medical records of patients	205 unrelated probands with various forms of cardiomyopathy were evaluated	Validates the impact of consanguinity on the genetics of childhood-onset cardiomyopathy

MERS, middle east respiratory syndrome; GDB, global burden of disease; IRFS, injuries and risk factors study; DALYs, disability adjusted life years; YLDs, years live with disability; NSCLC, non-small cell lung cancer; mRCC, metastatic renal cell carcinoma; CRC, colorectal cancer; HFpEF, heart failure with preserved ejection fraction; HFrFF, heart failure reduced ejection fraction.

### 3.2. Findings from the cross-sectional survey

Among the respondents, 61% identified as researchers, whereas 21% were associated with organizations that fund the health research projects. Participants were involved with various organizations directly and indirectly implicated in research work, and the majority were affiliated with the MoH (47%). Among the researchers, 28% were working as principal investigators and co-investigators (Table 3).

**Table 3:** Professional roles and affiliations of respondents (*n* = 384).

**Categories**	***f***	***%***
**Professional role**		
Researchers	233	61%
Administrator	71	18%
Representative of funding organization	80	21%
**Institutional affiliations**		
University/higher education institution	51	13%
Ministry of Health	182	47%
Ministry of Education	16	4%
Governmental hospitals	55	14%
Research funding organizations (e.g., Maarifah, R&D Grant program)	10	3%
Medical industry such as pharmaceutical industries, healthcare apps/medical equipment	59	15%
Consultant in research and development projects	11	4%
**Work role in current projects**		
Principal investigator/co-principal investigator	108	28%
Only co-investigator	10	3%
Consultant	97	25%
Administrator	106	27%
Representative of research funding organizations	63	17%

**Figure 2:**
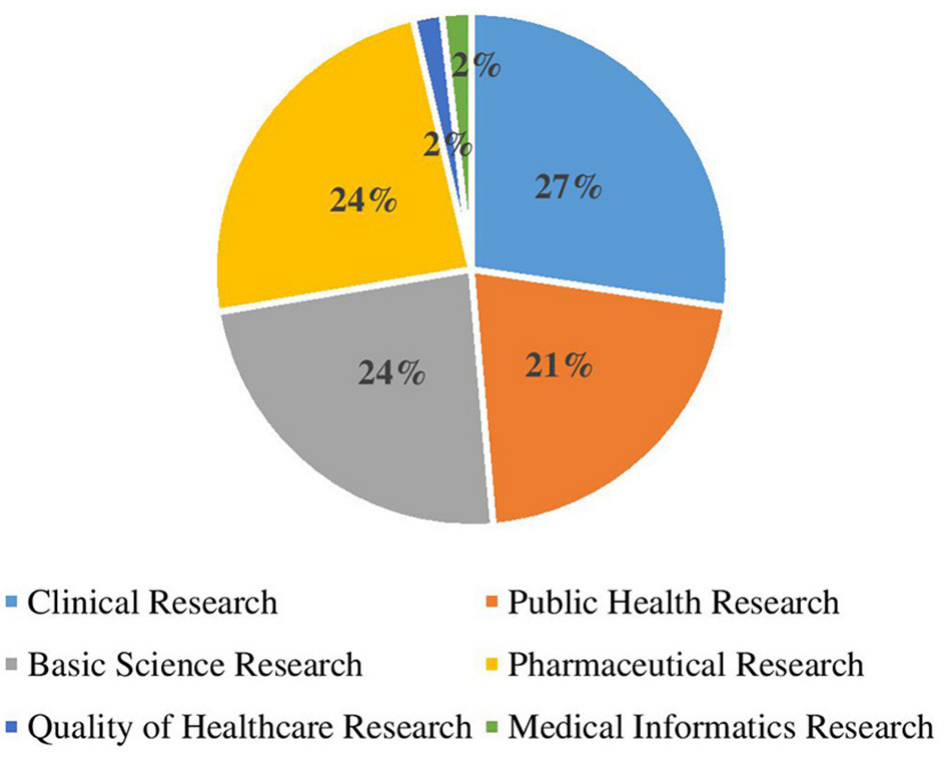
Broad areas of ongoing health research projects by respondents.

The analysis of ongoing projects of respondents revealed that almost one-third of the current research focuses on clinical sciences; almost one-quarter of the research is focused on basic sciences (24%), and an equal proportion is on pharmaceutical sciences (24%), and basic sciences (21%). The analysis revealed that around 2% of current research focuses on the quality of healthcare and (2%) on medical informatics (Figure 2).

The findings (Table 4) demonstrate among ongoing research projects carried out by respondents are on various diseases, included kidney and liver disorders (80%), obesity (74%), diabetes (74%), hormonal diseases (64%), infectious diseases (66%), orthopedic (56%), cardiac disorders (50%), respiratory diseases (47%), and digestive disorders (40%). The health issues that are less studied on in the current research projects include developmental disorders, both mental and physical disabilities, and psychiatric disorders; statistics show that only around (1%) of ongoing research projects by researchers have focused on these areas. Fewer ongoing research projects focuses on maternal healthcare, and less than one-tenth of research focuses on men and women's reproductive health disorders (Table 4).

**Table 4:** Health problems investigated in current research projects of respondents.

	***f***	**%**
**Health problems**		
Kidney and liver disorders	310	80%
Obesity disorders	287	74%
Diabetic diseases	286	74%
Infectious diseases	255	66%
Hormonal diseases	249	64%
Bone disorders/orthopedic	217	56%
Heart diseases/cardiac disorders	192	50%
Respiratory diseases	182	47%
Digestive disorders/gastroenterology	155	40%
Skin disease/dermatology	140	36%
Children's health/pediatrics	82	21%
Dental care and oral disorders	74	19%
Maternal healthcare/gynecology	17	7%
Vision/eye care/ophthalmology	16	4%
Hearing disorders/audiology	10	3%
Women's reproductive health disorders	11	3%
Men reproductive health disorders/urology	10	3%
Psychiatric disorders/psychological disorders	7	2%
Developmental physical disabilities (hearing, vision, and physical organs)	4	1%
Developmental mental disabilities (e.g., intellectual disability)	2	0.5%
**Other areas of medicine**		
Immunology	282	73%
Pharmaceutical sciences/drug trials	309	80%
Disease pathology	210	54%
Radiology/medical technology/medical informatics	113	29%
Surgery	68	17%
Intensive care of the seriously ill	4	1%
Life support and anesthesia	3	1%

**Figure 3:**
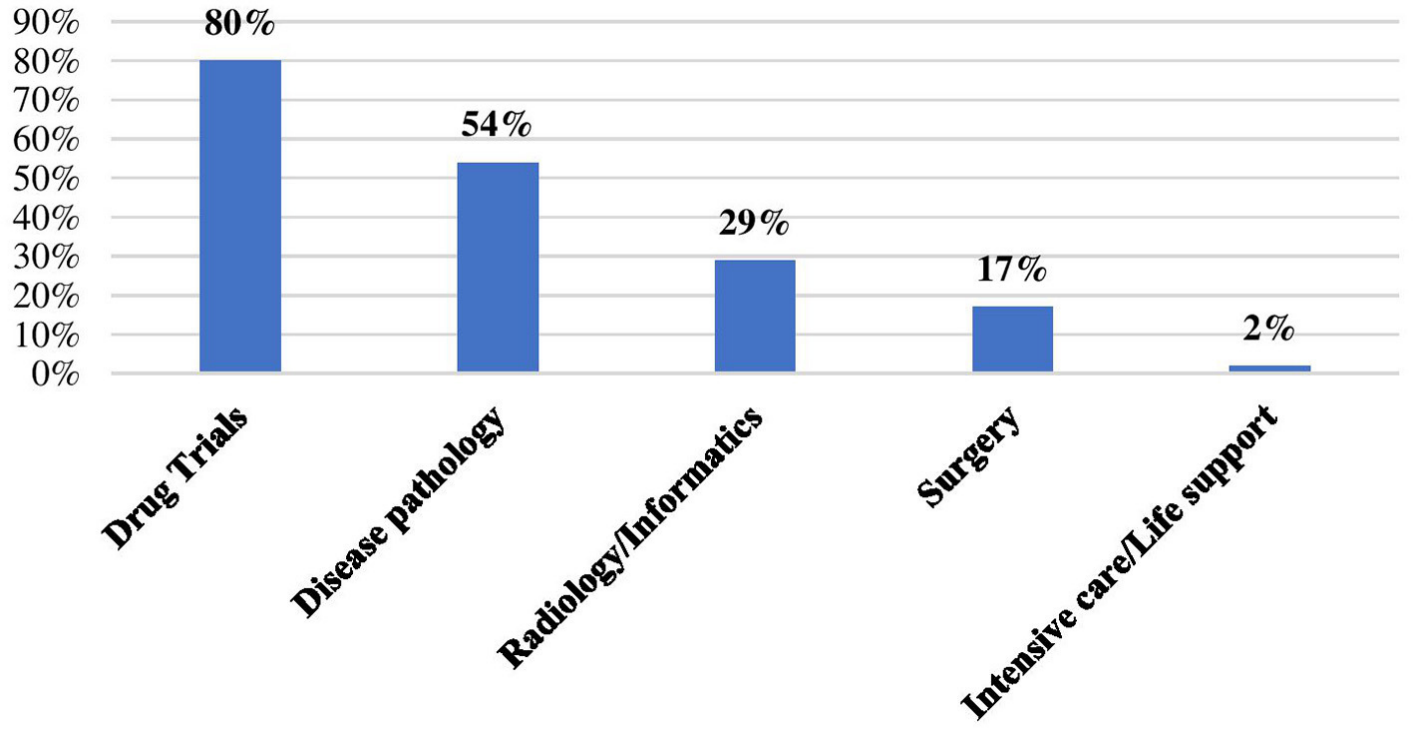
Specific areas of ongoing health research projects by respondents.

Among the specific areas of health research, 80% of current research projects are drug trials; over 50% of the ongoing research focuses on disease pathology (54%); nearly one-third of the studies focus on radiology and informatics (29%); and less than one-quarter focus on surgery (17%). Finally, few current research studies (2%) focus on intensive care and life support (Figure 3).

## 4. Discussion

The current study aimed to inspect the current focus, trends, and gaps in population health research in Saudi Arabia based on data obtained from a systematic review of high-impact journal publications in the past 2 years and a survey of research leaders to collect information about their ongoing research projects. The current study is significant because the Saudi government is moving toward a knowledge-based economy and has taken several initiatives over the past decade, such as research funding and collaborations with international research organizations for higher education, research and innovation (2). As a result, Saudi Arabia has experienced a significant increase in the number of publications in science journals. According to Saudi Gazette, Saudi Arabia ranked first in the Arab sphere in scientific research and 25th at the global level by the Scimago Index (13). It also reported an increase in the publication of scientific research to over 57,000 from local universities. Our study findings suggests that some of the important population health issues identified by the Global Burden of Diseases, Injuries, and Risk Factors Study (2017) (38) are the focus of ongoing research projects, and few original studies from Saudi Arabia were published in the top 50 Q1 journals in the field of medicine (Table 1). However, the protocol search also revealed that a substantial number of the articles published between January 2020 and January 2022 with affiliations of research organizations from universities in Saudi Arabia are based on meta-analyses and, systematic and non-systematic reviews of literature. The current study as well as previous literature (39) demonstrated the paucity of qualitative research that is published in high-impact medical research journals. There could be possibly different reasons for it such as the qualitative studies are time-consuming and are seen as less reliable evidence due to the subjective nature of the study. Moreover, it has been found that the rigor of qualitative research is considered as low because the sampling is usually based on convenience sampling and is less in number. These factors limit the generalizability of findings and discourage journals to publish qualitative research, which sometimes falls below the criterion used to assess the quality of research. After screening and evaluating, the population-based health studies that published and met the inclusion and exclusion criteria were limited to few. Thus, the current study strongly suggests the need to identify and address the challenges currently faced by researchers in conducting original studies and publishing research in the top high-impact journals. Lastly, there is need for a longitudinal analysis to determine the publication of qualitative research in high-impact medicine journals.

Our findings show that a smaller number of ongoing research projects have focused on maternal health, reproductive health, and developmental disabilities. This finding aligns with the previous literature (6, 38); which demonstrated that less funding and research resources are allocated for research projects in the area of maternal and infant health, and there is a need to address the existing gaps in the research and set these issues as priorities for future research. The findings also revealed that current research focuses less on studying psychological and behavioral problems, even though it has noticed a steady rise in years lived with a disability in Saudi Arabia due to psychiatric and substance abuse disorders between 2010 and 2017 (15). The authors of this study have noticed that several cross-sectional surveys were conducted during the COVID-19 pandemic to study its psychological and emotional repercussions (40, 41). However, to publish in the top Q1 journals, robustness must be adopted in research methodology and in reporting findings that are compromised due to limited resources to conduct and publish research. These challenges should be addressed to over-come the existing gaps in high-impact research in the health sciences.

Finally, the findings from the survey also demonstrated that a limited number of current projects study issues related to healthcare management and medical informatics. Since Saudi Arabia has adopted several health reforms and automation of healthcare systems, it is important to allocate more resources to study how this automation is increasing the efficiency of healthcare systems, as well as the challenges faced by the healthcare force and patients.

### 4.1. Study strengths, limitations, and directions for future research

This study adopted a mixed-method approach to conduct a comprehensive analysis of the current focus of scientific research on population health in Saudi Arabia. A systematic review of studies published in high-impact journals and a cross-sectional survey of key players in the field was an effective research methodology to accomplish the research objectives and provide useful insights. The current study's findings are helpful in identifying existing gaps in health research and making recommendations for future research priorities. The findings demonstrate some of the positive outcomes of initiatives taken by the Saudi MoH and MoE to promote scientific research in Saudi Arabia in the past decade, as a result, few important original studies were published in the top high-impact medical journals. Original scientific studies in the field of medicine require labs, technological equipment, and appropriate research training, along with the enhancement of critical thinking and creative abilities, to develop leaders in scientific research in Saudi Arabia. Therefore, the study findings underline the need to invest in these areas, and such measures will be effective in enhancing the outcomes of existing research initiatives and smooth progress to achieve the Saudi 2030 vision. Among the study limitations, the current systematic review limited the analysis to the specific period of the last 4 years and the top 50 high-impact journals. In addition, the cross-sectional survey comprised of close-ended questions and thus a detailed assessment of underlying reasons for the lack of research on certain topics were not conducted. Qualitative studies are needed to identify obstacles and issues that must be addressed to prioritize research areas. Qualitative studies are recommended in the future to obtain the opinions of leaders in scientific research, which will be useful in formulating strategic plans and addressing the existing gaps in health research. Understanding the needs and roles of stakeholders will provide more insight to overcome barriers and appropriate actions to achieve research and development goals, the ensure the availability of adequate resources at an institutional level to conduct such studies. Globally, research is being conducted at a fast pace, and it is necessary to design research that identifies, the specific reasons restricting the production and publication of high-impact health research in Saudi Arabia. Some of the priorities for future research funding in the health sciences in Saudi Arabia should focus on health issues such as physical and mental disabilities, reproductive health, maternal health and dental disorders. In additions, original studies should be funded to assess progress and development on health informatics and the quality of healthcare services in Saudi Arabia.

## 5. Conclusion

In conclusion, some of the important studies that have been conducted in the last decade on MERS-CoV, cancer and cardiac diseases in Saudi Arabia were published in few top high-impact science journals. This number must be increased further, and future research should focus on identifying the key reasons to address this gap. It is a positive development that most of the ongoing research projects focus on major health problems in the Saudi population such as kidney and liver diseases, obesity, diabetes and hormonal diseases. However, there are some existing gaps in research in some specific areas such as physical and mental disabilities, dental diseases and reproductive health problems should also be considered in future research. The impact of major developments in the healthcare industry, such as the expansion of health informatics systems and the delivery of healthcare services should also be investigated to devise appropriate healthcare policies and procedures.

## Data availability statement

The original contributions presented in the study are included in the article/supplementary material, further inquiries can be directed to the corresponding author.

## Ethics statement

The studies involving human participants were reviewed and approved by Ethical Review Committee University of Ha'il. The patients/participants provided their written informed consent to participate in this study.

## Author contributions

Conceptualization, systematic review, data analysis, write-up, and review and revision: AA-S. Systematic review, data analysis, write up, and review and revision: SH. Data collection, data extraction, and review and revision: FA. Systematic review, data extraction, review and revision, and references: MA. All authors contributed to the article and approved the submitted version.
